# The role of immune system associated genes in patients with Alzheimer’s disease: A large case‐control study in the Chinese population

**DOI:** 10.1002/alz.087849

**Published:** 2025-01-03

**Authors:** Xuewen Xiao, Bin Jiao, Rui Yao, Xinxin Liao, Jincheng Li, Beisha Tang, Lu Shen

**Affiliations:** ^1^ Xiangya Hospital, Central South University, Changsha, Hunan China

## Abstract

**Background:**

Various studies indicated that the immune system is a cardinal feature of Alzheimer’s disease (AD), which can either ameliorate or exacerbate AD neuropathogenesis. Nevertheless, the associations between genes involved in the immune system and AD remain unclear.

**Method:**

To systematically evaluate the associations of these genes with AD, we investigated 370 genes implicated in the immune system based on previous studies selected using the PubMed database. Our study recruited 1511 AD patients and 2001 cognitively normal controls. 370 genes were sequenced using a targeted panel. Variants were classified into common or rare variants with the minor allele frequencies (MAF) cutoff of 0.01. Common variant (MAF≥0.01)‐based association test was performed by PLINK 1.9, and gene‐based (MAF <0.01) association analysis was conducted using Sequence Kernel Association Test‐Optimal (SKAT‐O test) by combining rare variants between AD patients and cognitively normal controls. Rare variants were further categorized as followings: rare damaging variants (MAF <0.01, LoF or ReVe >0.7), rare damaging missense variants (MAF <0.01, ReVe >0.7), rare LoF variants (MAF <0.01, LoF), and rare missense variants (MAF <0.01, missense). Age, sex, and *APOE* ε4 status were adjusted in our study.

**Result:**

After quality control, 73,885 common variants were left. A common variant, *MGMT* rs147073440, was suggestively associated with AD risk after adjusting for age, sex, and *APOE* ε4 status and performing the Bonferroni correction {adjusted P = 1.62 × 10^‐6^, odds ratio (OR) [95% confidence interval (CI)] = 2.726 (1.809‐4.106)}. 250 genes with rare damaging variants were analyzed in our study. The gene‐based association analysis revealed that the rare damaging variants in the *RAB37* and *EPCAM* genes were nominally associated with AD (p <0.004).

**Conclusion:**

This study suggested that *MGMT* rs147073440 and rare damaging variants in *RAB37* and *EPCAM* may be implicated in AD pathogenesis.

